# Expression of specific ionotropic glutamate and GABA-A receptor subunits is decreased in central amygdala of alcoholics

**DOI:** 10.3389/fncel.2014.00288

**Published:** 2014-09-16

**Authors:** Zhe Jin, Amol K. Bhandage, Igor Bazov, Olga Kononenko, Georgy Bakalkin, Esa R. Korpi, Bryndis Birnir

**Affiliations:** ^1^Molecular Physiology and Neuroscience Unit, Neuroscience, Biomedical Center, Uppsala UniversityUppsala, Sweden; ^2^Division of Biological Research on Drug Dependence, Department of Pharmaceutical Biosciences, Uppsala UniversityUppsala, Sweden; ^3^Pharmacology, Institute of Biomedicine, University of HelsinkiHelsinki, Finland

**Keywords:** amygdala, GABA_A_ receptor, ionotropic glutamate receptors, brain, alcohol dependence, post-mortem

## Abstract

The central amygdala (CeA) has a role for mediating fear and anxiety responses. It is also involved in emotional imbalance caused by alcohol abuse and dependence and in regulating relapse to alcohol abuse. Growing evidences suggest that excitatory glutamatergic and inhibitory γ-aminobutyric acid-ergic (GABAergic) transmissions in the CeA are affected by chronic alcohol exposure. Human post-mortem CeA samples from male alcoholics (*n* = 9) and matched controls (*n* = 9) were assayed for the expression level of ionotropic glutamate and GABA-A receptors subunit mRNAs using quantitative real-time reverse transcription-PCR (RT-qPCR). Our data revealed that out of the 16 ionotropic glutamate receptor subunits, mRNAs encoding two AMPA [2-amino-3-(3-hydroxy-5-methyl-isoxazol-4-yl)propanoic acid] receptor subunits GluA1 and GluA4; one kainate receptor subunit GluK2; one NMDA (N-methyl-D-aspartate) receptor subunit GluN2D and one delta receptor subunit GluD2 were significantly decreased in the CeA of alcoholics. In contrast, of the 19 GABA-A receptor subunits, only the mRNA encoding the α2 subunit was significantly down-regulated in the CeA of the alcoholics as compared with control subjects. Our findings imply that the down-regulation of specific ionotropic glutamate and GABA-A receptor subunits in the CeA of alcoholics may represent one of the molecular substrates underlying the new balance between excitatory and inhibitory neurotransmission in alcohol dependence.

## Introduction

Alcoholism is a chronic relapsing brain disorder characterized by compulsive alcohol intake, progressive alcohol tolerance, dependence and behavioral impairments (Koob, 2013). Long-term excessive alcohol consumption has a lasting and negative impact on different brain regions, including the amygdala. A pronounced and early loss of amygdala neurons has been observed in both humans with alcohol dependence and chronically alcohol-fed rats (Alvarez et al., 1989). The amygdala is a key limbic structure associated with emotions including fear and stress responses. Importantly, a number of amygdala sub-regions are critical for alcohol reward, dependence and relapse (Koob and Le Moal, 2001; McBride, 2002; Cui et al., 2013). In particular, the central amygdala (CeA), the main component of the extended amygdala, participates in the neuroadaptation that takes place during development of alcohol dependence, such as mediation of alcohol self-administration and stress-induced reinstatement of alcohol seeking (Koob and Volkow, 2010; Roberto et al., 2012). The CeA is the major output station of the amygdala and contains mostly GABAergic projection neurons. It receives multiple afferent inputs from, e.g., the cortex and thalamus as well as glutamatergic input from the basolateral amygdala (BLA). The modulation of the dynamic balance between excitatory glutamatergic and inhibitory GABAergic neurotransmission in the CeA has been correlated with behavioral changes during chronic alcohol exposure and withdrawal (Kumar et al., 2009; McCool et al., 2010). For instance, bilateral CeA microinjection of a GABA-A receptor antagonist decreases alcohol self-administration (Hyytia and Koob, 1995), whereas CeA injection of ionotropic glutamate receptor antagonist inhibits alcohol-induced reward behavior in animals (Zhu et al., 2007).

As in other brain regions, both ionotropic glutamate receptors and GABA-A receptors in the CeA are ligand-gated multi-subunit ion channels. The ionotropic glutamate receptor is tetrameric and subdivided into four families of receptors, NMDA receptors (subunits GluN1, GluN2A-D, GluN3A-B), AMPA receptors (subunits GluA1-4), kainate receptors (subunits GluK1-5), and delta receptors (subunits GluD1-2) (Traynelis et al., 2010). The ionotropic glutamate receptors are permeable to cations and mediate excitatory synaptic transmission. However, it is unclear whether delta receptors can form functional channels (Schmid and Hollmann, 2008). The GABA-A receptors are pentameric GABA-activated anion channels, passing usually chloride (Cl^−^) ions, and generating phasic and tonic forms of inhibitory neurotransmission. To-date, 19 different mammalian GABA-A receptor subunits have been identified: α1-6, β1-3, γ1-3, δ, ε, θ, π, and ρ1-3 (Olsen and Sieghart, 2008). For both ionotropic glutamate and GABA-A receptors, a multitude of different subunits can assemble and potentially form a large number of receptor subtypes displaying distinctive physiological and pharmacological properties (Smart and Paoletti, 2012).

In the CeA, acute alcohol exposure generally inhibits ionotropic glutamate receptors (Zhu et al., 2007; McCool et al., 2010), but enhances GABAergic transmission via both pre- and postsynaptic mechanisms (Roberto et al., 2003, 2012; Herman et al., 2013). Neuroadaptations are observed in response to chronic alcohol exposure and affect both the glutamatergic and GABAergic transmission in the CeA as well as other brain regions (Boehm et al., 2004; McCool et al., 2010; Roberto et al., 2012; Lovinger and Roberto, 2013). Chronic alcohol exposure regulates spatial and temporal expression of ionotropic glutamate and GABA-A receptor subunit genes in the mammalian brain (Charlton et al., 1997; Hemby et al., 2006; Acosta et al., 2012; Jin et al., 2012, 2014). We recently showed in individuals with alcohol dependence that a number of ionotropic glutamate and GABA-A receptor subunit mRNAs are altered in the hippocampal dentate gyrus region in contrast to the orbitofrontal and dorsolateral prefrontal cortices, where expression of only a few or no subunit changed (Jin et al., 2012, 2014). In rats on 5% alcohol-containing liquid diet for 12 weeks, a significant reduction in the hippocampal mRNA level of the GABA-A α1 subunit was observed, whereas 4 weeks or less on the diet did not alter expression of the gene (Charlton et al., 1997). In alcohol-preferring AA rats, lifelong alcohol drinking (up to 24 months) was associated with a reduction in mRNA expression of the GABA-A receptor α4 and β4 subunit genes in several brain regions, but no alterations were observed in another 11 GABA-A receptor subunit genes studied, including the α1 (Sarviharju et al., 2006).

Animal models are important tools to study alcohol-related disorders, but they do not resemble all aspects of human alcohol addiction (Crabbe et al., 2013). Gene expression studies performed on human post-mortem brain tissues are, therefore, essential and provide valuable clues to mechanisms underlying alcohol-induced damage to the human brain. So far, the majority of human post-mortem brain studies have focused on cortical areas, and only a few reports have investigated amygdala subregions (He and Crews, 2008; Kuzmin et al., 2009; Kryger and Wilce, 2010).

In the present study, we performed reverse transcription quantitative PCR (RT-qPCR) to investigate the mRNA expression of ionotropic glutamate and GABA-A receptors subunits in the CeA samples from individuals with alcohol dependence and control subjects. The results show that the mRNA levels of 5 ionotropic glutamate receptor subunits (GluA1 and A4, GluK2, GluN2D, and GluD2) and the α2 GABA-A receptor subunit are significantly lower in human alcoholics as compared with control subjects.

## Materials and methods

### Human post-mortem samples

Post-mortem human brain samples from the CeA were obtained at the New South Wales Tissue Resource Center (TRC), University of Sydney, Australia (http://sydney.edu.au/medicine/pathology/trc/index.php). All samples were collected by qualified pathologists under full ethical clearance and with informed, written consent from the next of kin. All samples were immediately frozen and stored at −80°C until required for analysis. Nine human control subjects and nine individuals with chronic alcohol dependence were included in the study. All individuals were Caucasian males. Individuals in the control group were matched to individuals with chronic alcohol dependence by age and post-mortem interval (PMI). Individuals in the control group had either abstained from alcohol completely or were social drinkers who had consumed less than 20 g of alcohol per day on average. The individuals with chronic alcohol dependence had consumed more than 80 g alcohol per day for most of their adult lives, met the criteria for Diagnostic and Statistical Manual for Mental Disorders, 4th edition and National Health and Medical Research Council/World Health Organization and did not have liver cirrhosis, Wernicke-Korsakoff's syndrome or multi-drug abuse history. The detailed demographic characteristics for all subjects are summarized in Supplementary Table S1. There was no significant difference in age, PMI, brain pH, RQI and proportions of smokers and non-smokers between individuals with or without alcohol dependence (Supplementary Table S2).

### Total RNA extraction

Total RNAs were extracted by using RNeasy Lipid Tissue Mini Kit (QIAGEN, Maryland, USA) and quantified with a Nanodrop ND-1000 spectrophotometer (Nanodrop Technlogies, Inc., USA). The quality of RNA was determined by measuring RNA Quality Indicator (RQI) using Bio-Rad Experion (Bio-Rad Laboratories, Hercules, CA, USA) with Eukaryote Total RNA StdSens assay. RQI is equivalent to RNA integrity number (RIN) from Agilent (Denisov et al., 2008). RNA samples with RQI values greater than 5 are recommended for quantitative PCR studies (Fleige and Pfaffl, 2006). All samples included in this study had RQI more than 5. Average RQI of all samples was 6.64 ± 0.19 (mean ± s.e.m.) (78% of the samples had RQI greater than 6) indicating high quality of isolated total RNA.

### Quantitative real-time RT-PCR (RT-qPCR)

Total RNAs were reverse transcribed into cDNAs in a 20 μl reaction mixture using Superscript III reverse transcriptase (Invitrogen, USA). In order to confirm absence of genomic DNA contamination in the isolated RNA, the reverse transcriptase in the reaction was omitted which served as a negative control. The gene-specific primer pairs (primer sequences shown in Supplementary Table S3) were designed using NCBI Primer-BLAST and GETPrime (updepla1srv1.epfl.ch/getprime/) and validated with BioBank cDNAs from human brains (PrimerDesign Ltd., UK) by the identification of a single peak in the melting curve and a single band with the expected size on an agarose gel. Primer efficiency was not examined further as all PCR products were shorter than 200 base pairs. qPCRs were carried out in a 10 μl reaction mixture containing 4 μl cDNA, 1 × PCR reaction buffer, 3 mM MgCl_2_, 0.3 mM dNTP, 1 × ROX reference dye, 0.8 U JumpStart Taq DNA polymerase (Sigma-Aldrich, Germany), 5 × SYBR Green I (Invitrogen, USA) and 0.4 μM each of forward and reverse primers. Amplification was performed in 384-well optical plates using the ABI PRISM 7900HT Sequence Detection System (Applied Biosystems, USA) with an initial denaturation of 5 min at 95°C, followed by 45 cycles of 95°C for 15 s, 60°C for 30 s and 72°C for 30 s. A melting curve was obtained at the end of cycling to verify the amplification of a single PCR product. Cycle threshold values (Ct) were determined with the SDS 2.3 and RQ Manager 1.2 softwares supplied with the instrument. The expression of each target gene relative to a normalization factor (geometric mean of two reference genes) was calculated with DataAssist v2.0 using the 2^−ΔCt^ method as previously described (Schmittgen and Livak, 2008). Reference genes phosphoglycerate kinase 1 (PGK1) and TATA-binding protein (TBP) were used for normalization as previously described (Johansson et al., 2007; Kuzmin et al., 2009; Bazov et al., 2011).

### Statistical analysis

Statistical analysis was performed using Statistica (Statsoft Ltd., USA) and SigmaStat (Systat Software Inc., USA). Normality of data distribution was analyzed using Shapiro–Wilk normality test (See Supplementary Table S4). The differences between groups were assessed by One-Way ANOVA with Bonferroni *post-hoc* test (normal distributed data) or non-parametric Kruskal–Wallis ANOVA on ranks with Dunn's *post-hoc* test (non-normal distributed data). A general stepwise linear regression model was used to identify covariates (e.g., age and PMI). Variables with a significant association with group (controls and alcoholics) were included in the final statistical model as covariates. The significance level was set at *p* < 0.05.

## Results

The mRNA expression of 16 ionotropic glutamate receptor subunits (AMPA subunits: GluA1-4; kainate subunits: GluK1-5; NMDA subunits: GluN1, 2A, 2B, 2C, 2D, 3A and 3B; glutamate receptor delta: GluD1 and 2) and 19 GABA-A receptor subunits (α1-6, β1-3, γ1-3, δ, ε, θ, π, ρ1-3) was quantified by RT-qPCR in the CeA samples collected from nine control subjects and nine alcoholics.

### Decreased expression of five ionotropic glutamate receptor subunits mRNAs in the central amygdala of alcoholics

**Figure 1:**
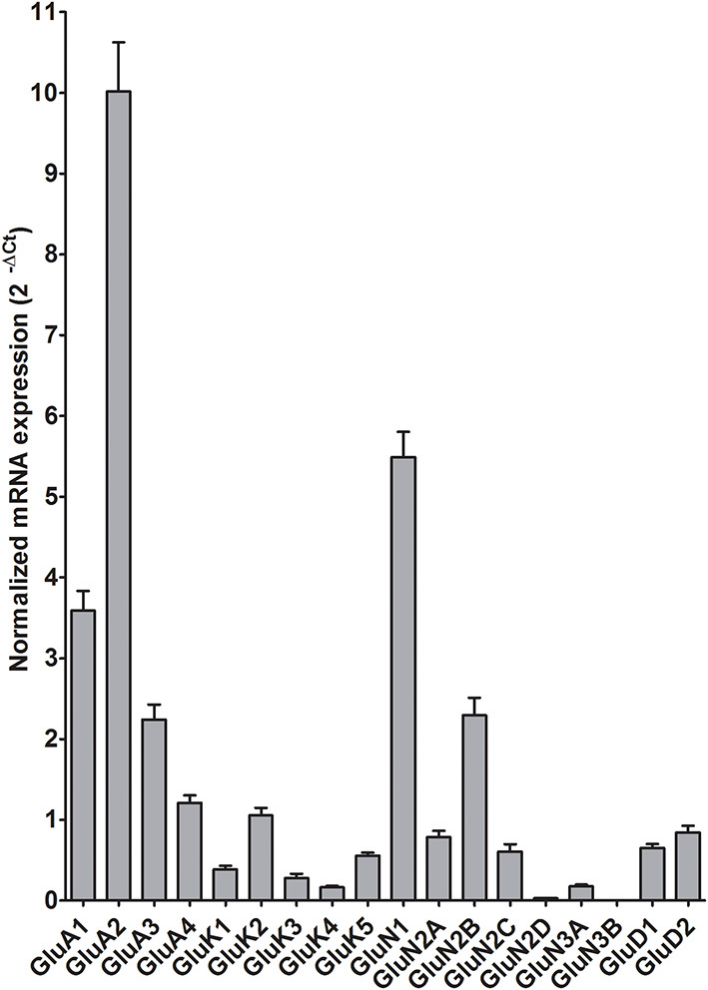
**Expression of ionotropic glutamate receptor subunit mRNAs in the central amygdala of control subjects (*n* = 9)**. The mRNA level of each subunit was normalized to reference genes PGK1 and TBP and presented as mean ± standard error of the mean (SEM).

In the CeA of individuals without alcohol dependence, there was high expression of GluA1, GluA2, and GluN1, modest expression of GluA3, GluA4, GluK2, and GluN2B, but lower expression of other ionotropic glutamate receptor subunit mRNAs (Figure 1). This qualitative estimation of high and modest mRNA expression levels was defined as equal to or great than that of GluA1 and GluK2, respectively.

**Figure 2:**
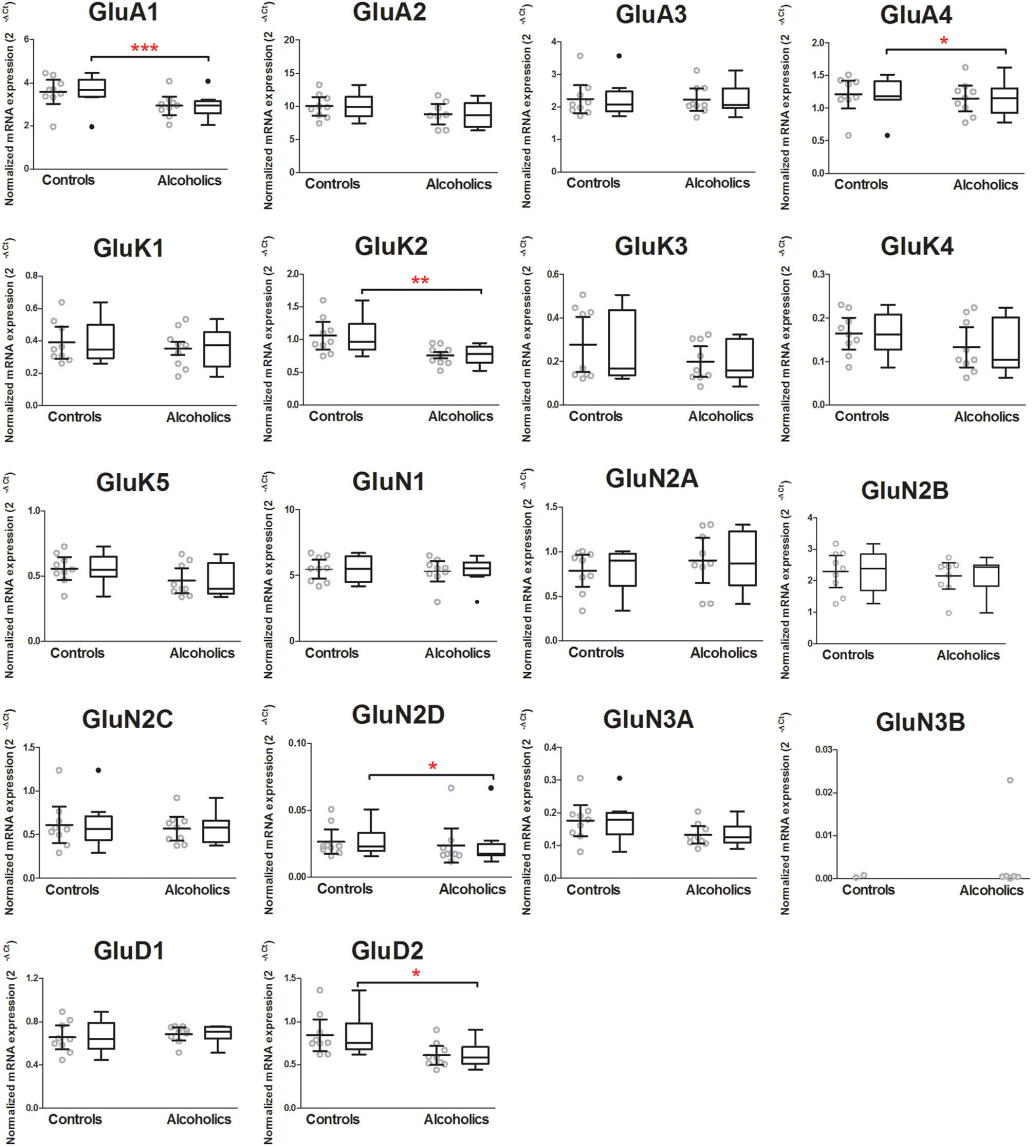
**Expression of ionotropic glutamate receptor subunits mRNAs in the central amygdala of controls (*n* = 9) and alcoholics (*n* = 9)**. Data from each group were presented as scatter dot plot (◦) with mean and 95% confidence interval and box and whiskers plot with median and whiskers plotted by Tukey method to determine outliers (•–above or below the whiskers). Outliers were excluded from the statistical analysis. One-Way ANOVA with Bonferroni *post-hoc* test: *GluA1*, *df* = 14, *p* = 0.00023; GluA2, *df* = 14, *p* = 0.15; GluA3, *df* = 14, *p* = 0.32; *GluA4*, *df* = 14, *p* = 0.04; GluK1, *df* = 14, *p* = 0.27; *GluK2*, *df* = 14, *p* = 0.0078; GluK4, *df* = 14, *p* = 0.22; GluK5, *df* = 14, *p* = 0.068; GluN1, *df* = 14, *p* = 0.99; GluN2A, *df* = 14, *p* = 0.38; GluN2B, *df* = 14, *p* = 0.43; GluN2C, *df* = 14, *p* = 0.76; GluN3A, *df* = 14, *p* = 0.12; GluD1, *df* = 14, *p* = 0.45. Kruskal–Wallis ANOVA on ranks with Dunn's *post-hoc* test: GluK3, *H*_(1, 18)_ = 1.22, *p* = 0.27; *GluN2D*, *H*_(1, 17)_ = 4.08, *p* = 0.043; *GluD2*, *H*_(1, 18)_ = 5.9, *p* = 0.015. ^*^p < 0.05; ^**^p < 0.01; ^***^p < 0.001.

Among all ionotropic glutamate receptor subunits, mRNA expression of AMPA receptor subunits GluA1 (74% of controls) and GluA4 (89% of controls), kainate receptor subunit GluK2 (72% of controls), NMDA receptor subunit GluN2D (69% of controls) and glutamate receptor delta GluD2 (72% of controls), was significantly lower in the CeA of alcoholics as compared to controls (Figure 2). Age, brain pH, PMI, smoking history, RQI or presence/absence of alcohol/benzodiazepines in the blood at death did not affect the significance between the two groups. GluN3B mRNA expression was only identified in 7 out of 18 individuals, and therefore the statistical analysis was not performed. There was no significant difference detected in mRNAs expression levels for the remaining ionotropic iGluR subunits between the two groups (Figure 2).

### Decreased expression of GABA-A receptor α2 mRNA in the central amygdala of alcoholics

**Figure 3:**
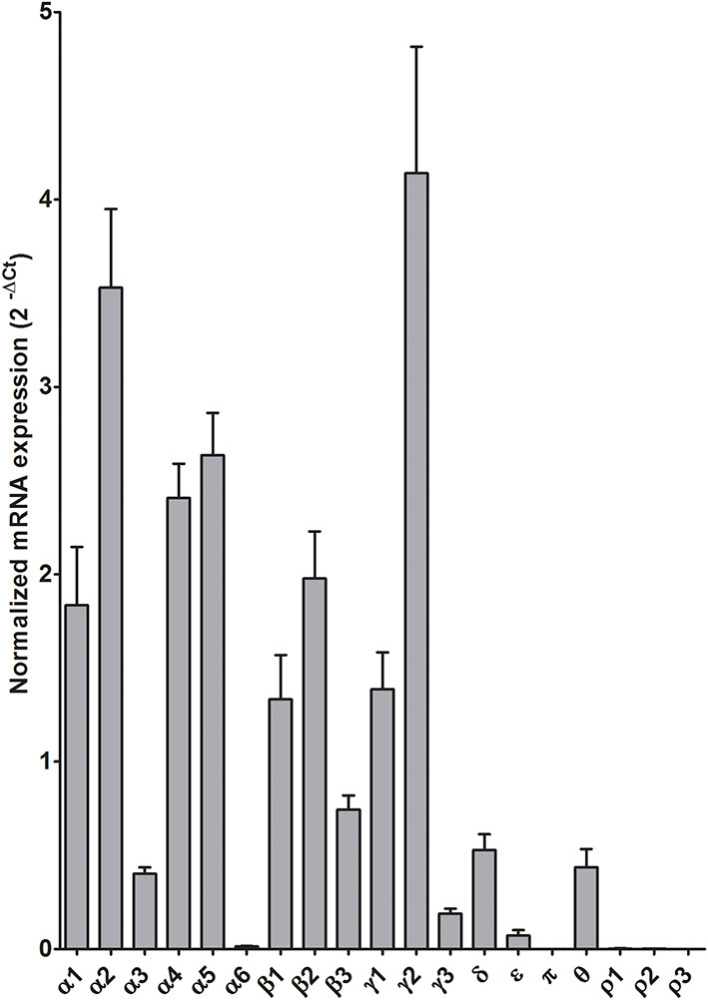
**Expression of GABA-A receptor subunit mRNAs in the central amygdala of control subjects (*n* = 9)**. The mRNA level of each subunit was normalized to reference genes PGK1 and TBP and presented as mean ± standard error of the mean (SEM).

In the CeA of individuals without alcohol dependence, high expression of α2, α4, α5, and γ2, modest expression of α1, β1, β2, and γ1, but lower expression of other GABA-A subunit mRNAs were detected (Figure 3). This qualitative estimation of high and modest mRNA expression levels was defined as equal to or great than that of α4 and β1, respectively.

**Figure 4:**
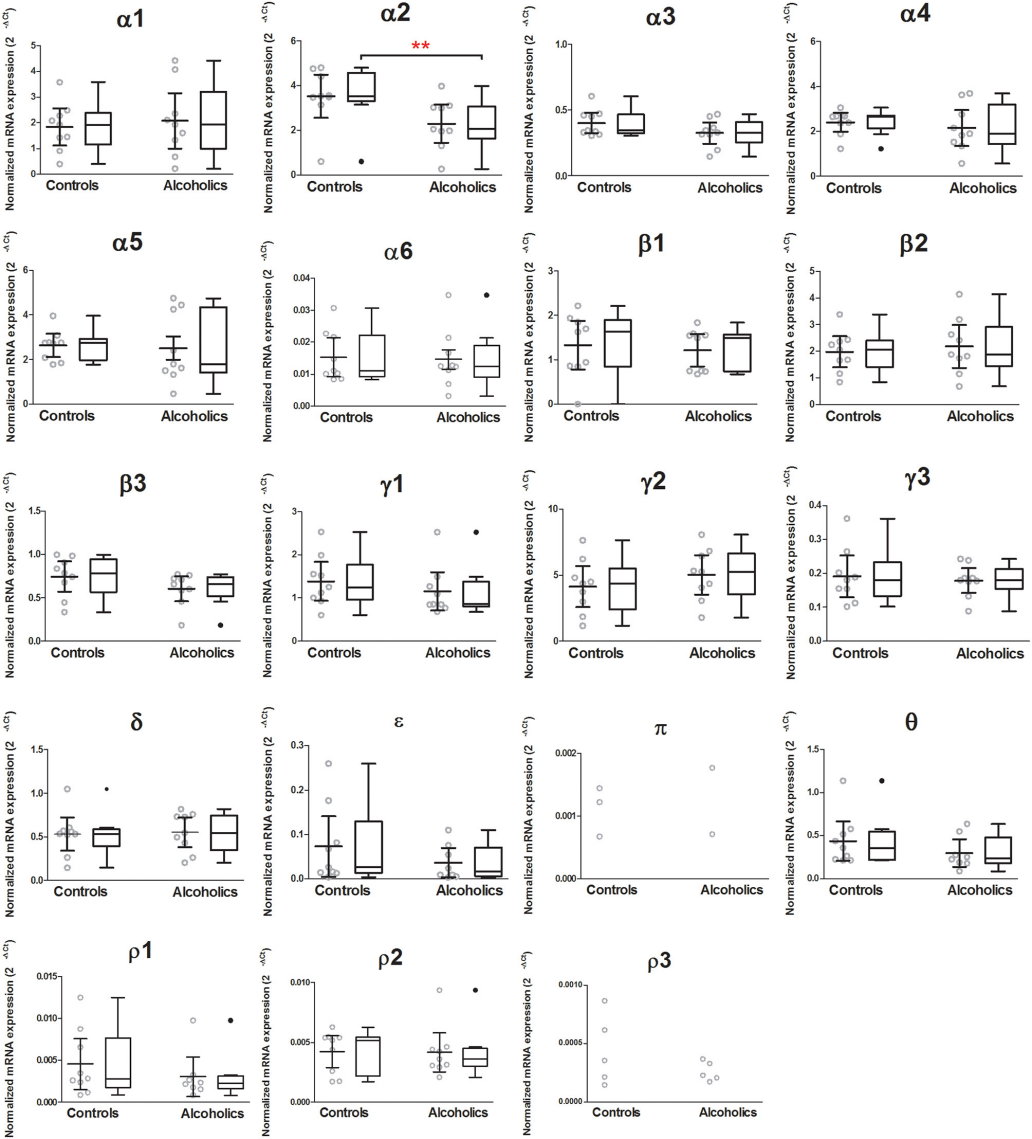
**Expression of GABA-A receptor subunit mRNAs in the central amygdala of controls (*n* = 9) and alcoholics (*n* = 9)**. Data from each group were presented as scatter dot plot (◦) with mean and 95% confidence interval and box and whiskers plot with median and whiskers plotted by Tukey method to determine outliers (•–above or below the whiskers). Outliers were excluded from the statistical analysis. One-Way ANOVA with Bonferroni *post-hoc* test: α1, *df* = 14, *p* = 0.61; α2, *df* = 14, *p* = 0.0029; α3, *df* = 14, *p* = 0.12; α4, *df* = 14, *p* = 0.26; α5, *df* = 14, *p* = 0.50; α6, *df* = 14, *p* = 0.25; β1, *df* = 14, *p* = 0.78; β2, *df* = 14, *p* = 0.61; β3, *df* = 14, *p* = 0.36; δ, *df* = 14, *p* = 0.33; γ2, *df* = 14, *p* = 0.36; γ3, *df* = 14, *p* = 0.88; ρ2, *df* = 14, *p* = 0.20; θ, *df* = 14, *p* = 0.55. Kruskal–Wallis ANOVA on ranks with Dunn's *post-hoc* test: ε, *H*_(1, 17)_ = 1.12, *p* = 0.29; γ1, *H*_(1, 17)_ = 3.00, *p* = 0.083; ρ1, *H*_(1, 16)_ = 2.36, *p* = 0.13. ^**^p < 0.01.

Out of 19 GABA-A receptor subunits, only the mRNA encoding the α2 subunit was significantly lower (59% of controls) in the CeA of alcoholics as compared to controls (Figure 4). Age, brain pH, PMI, smoking history, RQI or presence/absence of alcohol/benzodiazepines in the blood at death did not affect the significance between the two groups. Statistical analysis was not performed on subunits π and ρ3, as less than 60% of the samples expressed these 2 subunits. The mRNA levels of the remaining GABA-A subunits were not different between two groups (Figure 4).

## Discussion

The main finding of this study is a decrease in the expression of five ionotropic glutamate receptor subunits (GluA1, GluA4, GluK2, GluN2D, and GluD2) and one GABA-A receptor subunit (α2) in the CeA of human alcoholics. We have recently shown up-regulation of several subunit mRNAs of ionotropic glutamate (GluA2 and GluA3, GluK2, GluK3 and GluK5, GluN1, GluN2A, GluN2C, GluN2D and GluN3A) and GABA-A receptors (α1, α5, β1, and γ1) in the hippocampal dentate gyrus, up-regulation of GluN3A but down-regulation of GABA-A β2 and δ in the orbitofrontal cortex, and no difference in subunit expression in the dorsolateral prefrontal cortex of human alcoholics as compared to control subjects (Jin et al., 2012, 2014). Importantly, our studies and other gene expression studies on human post-mortem brain (Flatscher-Bader et al., 2006; Contet, 2012; Ponomarev et al., 2012), show that changes in gene expression pattern of the ionotropic glutamate and GABA-A receptors subunits induced by chronic alcohol intake greatly vary between brain regions.

There are various pre- and post-mortem factors, which may complicate the mRNA quantification in post-mortem human brain samples. We therefore assessed the potential factors such as age, brain pH, PMI, smoking history, quality of isolated mRNA and toxicology screening of the presence/absence of alcohol/benzodiazepines in the blood at death, and included the identified factors in the final statistical analysis as covariants. Our results show that none of these potential confounding factors affected the significance of differences in subunit mRNA expression between the alcoholic and control group.

The CeA integrates somatic and emotional information from other brain regions and other subregions within the amygdala (Pare and Duvarci, 2012). It plays an important role in motivation and stress of alcohol dependence (McBride, 2002). The majority of CeA neurons are GABAergic projection neurons, which receive glutamatergic excitatory inputs and locally connect to inhibitory interneurons. The functional neuroadaptations in glutamatergic and GABAergic system are essential for long-term effects of chronic alcohol consumption in the CeA of rodents (Roberto et al., 2003, 2004, 2012), which are often mirrored by alterations in the gene expression of receptor subunits (Roberto et al., 2006; Freeman et al., 2013). Our findings complement and extend previous studies in rodent models and confirm the impact of chronic alcohol exposure on the ionotropic glutamate and GABA-A receptors subunits mRNAs expression of in the CeA in humans.

Among the four families of ionotropic glutamate receptors, NMDA receptors are by far most frequently associated with alcohol effects in the CeA. NMDA receptor subunits are encoded by 7 genes, known as GluN1, GluN2A-D, and GluN3A-B. Genetic variants in GluN2A are strongly associated with human alcohol dependence (Schumann et al., 2008). Functional NMDA receptors are ion channels composed of 2 GluN1 subunits plus either 2 GluN2 subunits or a combination of GluN2 and GluN3 subunits, mediating the slow component of excitatory neurotransmission and contributing to synaptic plasticity (Smart and Paoletti, 2012). Our findings provide the first evidence of differential expression of NMDA receptor subunits in the adult human CeA. Six out of seven subunit gene mRNAs (GluN1, GluN2A-2D, and Glu3A) were consistently detected in total RNA samples prepared from CeA of 9 male control subjects, whereas GluN3B mRNA was only observed in 3 control samples. If one assumes PCR reactions for all subunits have equivalent efficiencies, the relative abundance of NMDA receptor subunit mRNAs in human CeA would be GluN1>GluN2B>GluN2A=GluN2C>GluN3A≫GluN2D>GluN3B, which is slightly different from that shown in the rat CeA GluN1=GluN2B>GluN2A≫GluN2C=GluN3A>GluN3B>GluN2D (Lack et al., 2005). However, the precise distribution of the 7 NMDA receptor subunits across CeA subdivisions (lateral and medial CeA) and different subtypes of CeA neurons (GABAergic projection neurons and interneurons) requires further investigation. *In vitro* electrophysiological studies have shown that 2 weeks of alcohol exposure to rats does not change the synaptic function of CeA NMDA receptors (Roberto et al., 2004), although it increases the levels of GluN1 and GluN2B subunit mRNAs in the CeA (Roberto et al., 2006). Interestingly, Lack et al. (2005) have shown that there were no significant changes in expression of any of the NMDA subunit mRNAs and proteins in rat CeA after 10–12 days exposure to alcohol. In addition, a more modest chronic exposure (at least 35 days) to an alcohol-containing liquid diet only mildly decreased the mRNA expression of GluN2C and GluN3A in the rat CeA (Freeman et al., 2013). One previous study performed on human post-mortem cortical samples has shown that the mRNA expression of GluN1, GluN2A, and GluN2B did not differ between controls and non-co-morbid alcoholics (Ridge et al., 2008). Similarly, our results show that in the CeA, the mRNAs of NMDA receptor subunits with the exception of GluN2D do not differ between controls and alcoholics. Due to the rather low expression of GluN2D in comparison to the other NMDA receptor subunits, its reduced expression in the CeA of human alcoholics may not affect glutamate receptor signaling in the CeA. Taken together, chronic alcohol-induced expression changes of NMDA receptor subunits in the CeA are likely dependent on multiple factors such as species, exposure pattern, dose and duration, and involves transcriptional regulation.

The effects of chronic alcohol exposure on non-NMDA (AMPA, kainate and delta receptors) have been less well characterized in the CeA. Our results show down-regulation of non-NMDA ionotropic glutamate receptor subunits (GluA1 and GluA4, GluK2 and GluD2) in the alcoholics, suggesting altered glutamatergic signaling in the CeA. Indeed, it has been shown that chronic alcohol treatment reduced non-NMDA receptor-mediated excitatory synaptic transmission (Roberto et al., 2004) and decreased the mRNA expression level of AMPA receptor subunits GluA2 and GluA3 in the rat CeA (Freeman et al., 2013). A recent study has shown that the change of GluA1 expression in the CeA modulates the associative learning of context-drug reward (Cai et al., 2013), consistent with a role in the development of alcohol dependence. The association of CeA kainate and delta receptors with chronic alcohol effects remains to be determined. Hippocampal excitatory kainate receptors on GABAergic interneurons have been shown to be very sensitive to ethanol antagonism (Carta et al., 2003), revealing a disinhibitory effect of ethanol on principal neurons. Recently, a single nucleotide polymorphism of GluK1 gene (*GRIAK1*) in the 3′ untranslated intronic region has been suggested to associate with alcohol dependence and shown to affect the anti-alcoholic treatment effects of topiramate that is a non-NMDA glutamate receptor antagonist (Kranzler et al., 2009, 2014). However, expression of this subunit was very low in the CeA of our samples and similar between alcoholics and controls (Figures 1, 2).

Alterations in GABAergic function in the CeA contribute to the development of alcohol dependence (Roberto et al., 2012). For example, microinjection of GABA agonist/antagonist into the CeA changes alcohol self-administration behavior in alcohol-dependent rats (Hyytia and Koob, 1995; Roberts et al., 1996). On the other hand, alcohol itself can modulate GABAergic transmission in the CeA. *In vitro* electrophysiological results show that acute application of alcohol increases GABA-A mediated inhibitory postsynaptic potentials (IPSPs) and currents (IPSCs) as well as the frequency and amplitude of spontaneous miniature IPSCs in rat CeA neurons, demonstrating the enhancement of GABAergic neurotransmission by alcohol at both pre- and postsynaptic sites (Roberto et al., 2003). A recent study suggests that alcohol enhances the tonic conductance mediated by α1 or δ subunit containing GABA-A receptors in rat CeA neurons (Herman et al., 2013). However, we did not find any changes of α1 or δ subunit mRNAs in the CeA of human alcoholics. The GABA-A α2 subunit at least partly mediates the anxiolytic and rewarding actions of benzodiazepines (Low et al., 2000; Engin et al., 2014) and in alcohol-preferring rats, inhibition of the α2 subunit expression causes profound reduction of binge alcohol drinking (Liu et al., 2011). In rodents, the α2 subunit is the main α subunit in the CeA (Marowsky et al., 2004), and similarly, in our expression profiling it emerged as the most abundant α subunit in the human CeA (Figure 3). Our results show a significant decrease of the α2 subunit mRNA in the CeA of human alcoholics, which reflects adaptation in gene expression caused by chronic alcohol consumption. In addition, human genetic linkage studies show a strong association of *GABRA2* (the gene encoding the α2 subunit) with alcohol dependence (Edenberg et al., 2004; Haughey et al., 2008; Soyka et al., 2008; Roh et al., 2011; Villafuerte et al., 2012). These findings support the notion of an important role for the α2 subunit in the neuroadaptation in the CeA associated with chronic alcohol exposure. There are four major alternative splicing variants of the human *GABRA2* gene expressed in human brains (Tian et al., 2005). The primers used to detect α2 subunit expression in our study are not splice variant-specific and amplify all four isoforms in the CeA samples. It will be intriguing to further investigate whether all or specific *GABRA2* splice variants are down-regulated in the CeA of human alcoholics. The PCR fragment amplified with our *GABRA2* primers covers the region between exon 6 and 7, which does not have any common *GABRA2* SNPs (single nucleotide polymorphisms) associated with alcohol dependence (Edenberg et al., 2004). Therefore, the decreased expression of α2 subunit mRNA in the CeA of human alcoholics is not due to reduced binding affinity of our primer caused by alcoholism-enriched SNP variants. However, it would be of interest to know whether any of the alcoholics in this study had mutant alleles resulting in altered *GABRA2* expression from those with wild-type alleles.

Quantitative neuropathological studies have shown volume reduction and neuronal loss in the basolateral/lateral amygdala of human alcoholics (Alvarez et al., 1989; Wrase et al., 2008). However, to the best of our knowledge, no previous neuropathological data have been published on the CeA of human alcoholics. Therefore, it is unclear whether neuronal loss or altered metabolism or both, induced by chronic alcohol consumption, contributes to the reduced expression of the specific ionotropic glutamate and GABA-A receptor subunits mRNAs in the CeA of human alcoholics in our study. It is possible that changes in one of the neurotransmitter system drives changes in the other in order to maintain neurons functioning within their dynamic range (Yizhar et al., 2011; Remme and Wadman, 2012). Interestingly, when GABA-A receptors were ectopically expressed in mouse hippocampal neurons, adjustments in glutamate receptors were observed (Moykkynen et al., 2007).

In conclusion, the specific ionotropic glutamate and GABA-A receptor subunits in the CeA of individuals with alcohol dependence represent molecular substrates altered during the course of alcohol dependence. The down-regulation of the receptor expression and the resulting new balance between the excitatory and inhibitory neurotransmission implies modified amygdala's activity in alcoholics. Together with our earlier findings, our present data suggest brain region- and neuronal circuitry-dependent modulation of the expression of glutamate and GABA receptors in alcoholism that might underlie the difficulties in developing efficient pharmacological therapies.

## Author contributions

Igor Bazov, Olga Kononenko, Georgy Bakalkin obtained the material and made the RNA from the tissue, Zhe Jin and Amol K. Bhandage designed primers and ran the qPCR, Zhe Jin, Esa R. Korpi, and Bryndis Birnir designed the experiments. Amol K. Bhandage and Zhe Jin made the figures and did the statistical analysis, Zhe Jin and Bryndis Birnir wrote the paper that was edited by Esa R. Korpi and then commented on by other authors.

### Conflict of interest statement

The authors declare that the research was conducted in the absence of any commercial or financial relationships that could be construed as a potential conflict of interest.
