# Supplementary material for: Expression of specific ionotropic glutamate and GABA-A receptor subunits is decreased in central amygdala of alcoholics
Source: Front Cell Neurosci. 2014 Sep 16;8:288. doi: 10.3389/fncel.2014.00288 (PMC4165314; doi:10.3389/fncel.2014.00288)
Supplement: Supplementary file 1 [file Table1.DOCX]

**Table S1.** Demographic data of controls and alcoholics.

| **Subject**  **No.** | **Age**  **(Years)** | **PMI**  **(Hours)** | **Brain pH** | **Smoking**  **history** | **RQI** | **Cause of Death** | **Toxicology screen of blood alcohol/benzodiazepines at death** |
| --- | --- | --- | --- | --- | --- | --- | --- |
| **CONTROLS** | | |  |  |  |  |  |
| **1** | 34 | 20.5 | 6.73 | Yes | 5.5 | Acute exacerbation of asthma | No |
| **2** | 63 | 72 | 6.9 | Ex-smoker | 7.5 | Severe coronary artery atherosclerosis | No |
| **3** | 82 | 23.5 | 6.4 | NA | 5.7 | Multiple organ failure-sepsis due to small bowel perforation |  |
|  |  |  |  |  |  |  | No |
| **4** | 69 | 16 | 6.6 | Yes | 6.2 | Atherosclerotic cardiovascular disease | No |
| **5** | 56 | 24 | 6.53 | Yes | 7 | Cardiac coronary artery atheroma | No |
| **6** | 59 | 20 | 6.56 | Yes | 7.1 | Coronary thrombosis | No |
| **7** | 56 | 37 | 6.76 | Yes | 6.4 | Left ventricular scarring , hypertension and cardiomegaly |  |
|  |  |  |  |  |  |  | No |
| **8** | 44 | 50 | 6.6 | Ex-smoker | 6.8 | Ischaemic heart disease | No |
| **9** | 53 | 16 | 6.84 | No | 8.1 | Dilated cardiomyopathy | No |
| **ALCOHOLICS** | | |  |  |  |  |  |
| **1** | 34 | 8.5 | 6.61 | Yes | 8.1 | Hanging | Yes |
| **2** | 77 | 20 | 6.34 | Yes | 5.5 | Bronchopneumonia | No |
| **3** | 39 | 24 | 6.56 | Yes | 6.3 | Aortic stenosis | NA |
| **4** | 56 | 45 | 6.51 | NA | 7 | Bleeding oesophageal varices | Yes |
| **5** | 56 | 22 | 6.52 | Yes | 6.2 | Coronary artery disease and upper gastrointestinal haemorrhage |  |
|  |  |  |  |  |  |  | No |
| **6** | 56 | 15 | 6.66 | NA | 6.4 | Ischaemic heart disease and emphysema | Yes |
| **7** | 81 | 36 | 6.44 | Ex-smoker | 5.9 | Sepsis | No |
| **8** | 44 | 15 | 6.48 | No | 7.3 | Ischaemic heart disease | Yes |
| **9** | 53 | 60 | 6.75 | Yes | 6.6 | Chronic airflow limitation | NA |

PMI, post-mortem interval; NA, not available. All of them were Caucasian males.

**Table S2.** Sample demographic information.

| **Characteristics** | **Amygdala** | | |
| --- | --- | --- | --- |
|  | **Controls** | **Alcoholics** | ***p* Value** |
| Number | 9 | 9 |  |
| Age (years) | 57±5 | 55±5 | 0.75 |
| PMI (h) | 31±6.3 | 27±5.5 | 0.54 |
| Brain pH | 6.7±0.05 | 6.5±0.04 | 0.10 |
| RNA quality indicator | 6.7±0.27 | 6.6±0.26 | 0.71 |
| Smoking history* | 7(87.5%) S, 1(12.5%)NS  Available:8 | 6(86%) S, 1(14%) NS  Available:7 | 1.00 |

PMI, post-mortem interval; S,smoker; NS,non-smoker. Age, PMI, brain pH, and RNA quality indicator are shown as mean ± SE, and the difference between controls and alcoholics was tested with Student’s t-test or Mann–Whitney U-test.

*Smoking histories are not available for all subjects. The proportion of smokers and non-smokers between controls and alcoholics was tested with Fisher’s exact test.

**Table S3.** Primers list for RT-qPCR

| **Genes** | **Accession no.** | **FD Primer Sequence** | **RV Primer Sequence** | **Amplicon Size (bp)** |
| --- | --- | --- | --- | --- |
| *GABRA1* (α1) | NM_000806.5 | GTCACCAGTTTCGGACCCG | AACCGGAGGACTGTCATAGGT | 119 |
| *GABRA2* (α2) | NM_000807.1 | GTTCAAGCTGAATGCCCAAT | ACCTAGAGCCATCAGGAGCA | 160 |
| *GABRA3* (α3) | NM_000808.3 | CAACTTGTTTCAGTTCATTCATCCTT | CTTGTTTGTGTGATTATCATCTTCTTAGG | 102 |
| *GABRA4* (α4) | NM_000809.2 | TTGGGGGTCCTGTTACAGAAG | TCTGCCTGAAGAACACATCCA | 105 |
| *GABRA5* (α5) | NM_000810.2 | TTGGATGGCTACGACAACAGA | GTCCTCACCTGAGTGATGCG | 62 |
| *GABRA6* (α6) | NM_000811.2 | ACCCACAGTGACAATATCAAAAGC | GGAGTCAGGATGCAAAACAATCT | 67 |
| *GABRB1* (β1) | NM_000812.2 | TGCATGTATGATGGATCTTCG | GTGGTATAGCCATAACTTTCGA | 80 |
| *GABRB2* (β2) | NM_021911.2 | GCAGAGTGTCAATGACCCTAGT | TGGCAATGTCAATGTTCATCCC | 137 |
| *GABRB3* (β3) | NM_000814.4 | CAAGCTGTTGAAAGGCTACGA | ACTTCGGAAACCATGTCGATG | 108 |
| *GABRD* (δ) | NM_000815.2 | CTTTGCTCATTTCAACGCC | TTCCTCACGTCCATCTCTG | 86 |
| *GABRE* (ε) | NM_004961.3 | ACAGGAGTGAGCAACAAAACTG | TGAAAGGCAACATAGCCAAA | 107 |
| *GABRG1* (γ1) | NM_173536.3 | CCTTTTCTTCTGCGGAGTCAA | CATCTGCCTTATCAACACAGTTTCC | 91 |
| *GABRG2* (γ2) | NM_000816.2 | CACAGAAAATGACGGTGTGG | TCACCCTCAGGAACTTTTGG | 136 |
| *GABRG3* (γ3) | NM_033223.3 | AACCAACCACCACGAAGAAGA | CCTCATGTCCAGGAGGGAAT | 113 |
| *GABRP* (π) | NM_014211.2 | CAATTTTGGTGGAGAACCCG | GCTGTCGGAGGTATATGGTG | 110 |
| *GABRR1* (ρ1) | NM_002042.3 | Hs00266687_m1 from Applied Biosystem |  | 94 |
| *GABRR2* (ρ2) | NM_002043.2 | TACAGCATGAGGATTACGGT | CAAAGAACAGGTCTGGGAG | 81 |
| *GABRR3* (ρ3) | NM_001105580.1 | TGATGCTTTCATGGGTTTCA | CGCTCACAGCAGTGATGATT | 111 |
| *GABRQ* (θ) | NM_018558.1 | CCAGGGTGACAATTGGCTTAA | CCCGCAGATGTGAGTCGAT | 63 |
| *GRIA1* (GluA1) | NM_000827 | ACTGGAAGAGACCCAAGTACACCTC | AGACAATCCCCAGCATTCCCCC | 127 |
| *GRIA2* (GluA2) | NM_000826 | CGCAGTCACTAATGCTTTCTGCTCC | AGGAGACGTGGAGTGTTCCGCA | 119 |
| *GRIA3* (GluA3) | NM_000828 | ACACCATCAGCATAGGTGGA | CTTCTCGGTGGTGTTCTGGT | 107 |
| *GRIA4* (Glu42) | NM_000829 | GGACACTCAAACAGGTTCG | TTGTGTAATTGACTCTACGTCC | 80 |
| *GRIK1* (GluK1) | NM_000830 | AGCAACAAAGACAAGTCCA | GGGTTCTTCCAGAATGGTG | 81 |
| *GRIK2* (GluK2) | NM_021956 | AAAGTGGTCGATGGAACGA | TAGAGCAGCATCAGTCGTC | 85 |
| *GRIK3* (GluK3) | NM_000831 | AGAGAGCAGCGTTCCTTCTG | CGGCGGTCATTGAA TGTGT | 146 |
| *GRIK4* (GluK4) | NM_014619 | TGAGGATCGCTGCTATCTTGG | CGTACTCGCTGTCTCTGAGAA | 159 |
| *GRIK5* (GluK5) | NM_002088 | CAGGTGCTCTCATCACTGCG | CTGACACATGGTGTCCGTGGT | 198 |
| *GRIN1* (GluN1) | NM_000832 | CATCCTCAAGTCCCACGAG | TTCCTGATACCGAACCCAC | 67 |
| *GRIN2A* (GluN2A) | NM_000833 | GAATGATCGGTGAAGTGGTC | CCACTTCAGAACGTTCCTC | 81 |
| *GRIN2B* (GluN2B) | NM_000834 | GGCAGATAAGGATGAATCCTC | ATGATGTTGAGCATTACGGA | 81 |
| *GRIN2C* (GluN2C) | NM_000835 | CTTCTTGGAGCCATATAGCC | AACATGAAGACGGTGATGG | 85 |
| *GRIN2D* (GluN2D) | NM_000836 | ATGGTTTCCTTCCTGAGCT | ATGAAGTACCTATGCAGACTCTC | 82 |
| *GRIN3A* (GluN3A) | NM_133445 | GCCATAGAAGGATACGGCA | CTTGTATTGACTGATTAGCTCGG | 81 |
| *GRIN3B* (GluN3B) | NM_138690 | CCTGTCCGAGTTCATCAG | CTGCAGGGTCTCTGTAAC | 118 |
| *GRID1* (GluD1) | NM_017551 | TGGTGACTGTCTTGGAAGAG | TGGAGAACCCTTTGTAGCG | 81 |
| *GRID2* (GluD2) | NM_001510 | TTATCCTAAGAGTGGTCACAGAG | GTATTCCACGGATATCGTATTCAC | 81 |
| *PGK1* | NM_000291.3 | AGGGAAAAGATGCTTCTGGG | AAGTGAAGCTCGGAAAGCTTCTAT | 71 |
| *TBP* | NM_003194.3 | GAGCTGTGATGTGAAGTTTCC | TCTGGGTTTGATCATTCTGTAG | 117 |

**Table S4.** Analysis of normality of RT-qPCR data distribution by Shapiro–Wilk normality test.

|  | **Amygdala** |
| --- | --- |
| α1 | = 0.3 |
| α2 | = 0.43 |
| α3 | = 0.23 |
| α4 | = 0.8 |
| α5 | = 0.43 |
| α6 | = 0.17 |
| β1 | = 0.18 |
| β2 | = 0.72 |
| β3 | = 0.8 |
| δ | = 0.27 |
| ε | < 0.05 |
| γ1 | < 0.05 |
| γ2 | = 0.89 |
| γ3 | = 0.11 |
| π | NA |
| ρ1 | < 0.05 |
| ρ2 | = 0.45 |
| ρ3 | NA |
| θ | = 0.061 |
| GluA1 | = 0.99 |
| GluA2 | = 0.9 |
| GluA3 | = 0.055 |
| GluA4 | = 0.98 |
| GluK1 | = 0.66 |
| GluK2 | = 0.13 |
| GluK3 | < 0.05 |
| GluK4 | = 0.25 |
| GluK5 | = 0.27 |
| GluN1 | = 0.53 |
| GluN2A | = 0.35 |
| GluN2B | = 0.54 |
| GluN2C | = 0.81 |
| GluN2D | < 0.05 |
| GluN3A | = 0.46 |
| GluN3B | NA |

p<0.05 indicates the data are not normally distributed, NA: not available because gene expression was only detected in few samples.
